# Targeting hexokinase 2 enhances response to radio-chemotherapy in glioblastoma

**DOI:** 10.18632/oncotarget.11680

**Published:** 2016-08-29

**Authors:** Alenoush Vartanian, Sameer Agnihotri, Mark R. Wilson, Kelly E. Burrell, Peter D. Tonge, Amir Alamsahebpour, Shahrzad Jalali, Michael S. Taccone, Sheila Mansouri, Brian Golbourn, Kenneth D. Aldape, Gelareh Zadeh

**Affiliations:** ^1^ MacFeeters Hamilton Center for Neuro-Oncology, Toronto, Canada; ^2^ Arthur and Sonia Labatt Brain Tumour Research Centre, SickKids Hospital, Toronto, Canada; ^3^ University Health Network, Toronto Western Hospital, Toronto, Canada

**Keywords:** glioblastoma, metabolism, cell signaling, novel treatments

## Abstract

First-line cancer therapies such as alkylating agents and radiation have limited survival benefits for Glioblastoma (GBM) patients. Current research strongly supports the notion that inhibition of aberrant tumor metabolism holds promise as a therapeutic strategy when used in combination with radiation and chemotherapy. Hexokinase 2 (HK2) has been shown to be a key driver of altered metabolism in GBM, and presents an attractive therapeutic target. To date, no study has fully assessed the therapeutic value of targeting HK2 as a mechanism to sensitize cells to standard therapy, namely in the form of radiation and temozolomide (TMZ). Using cell lines and primary cultures of GBM, we showed that inducible knockdown of HK2 altered tumor metabolism, which could not be recapitulated by HK1 or HK3 loss. HK2 loss diminished both *in vivo* tumor vasculature as well as growth within orthotopic intracranial xenograft models of GBMs, and the survival benefit was additive with radiation and TMZ. Radio-sensitization following inhibition of HK2 was mediated by increased DNA damage, and could be rescued through constitutive activation of ERK signaling. This study supports HK2 as a potentially effective therapeutic target in GBM.

## INTRODUCTION

Glioblastoma (GBM) is the most common and malignant primary brain tumor, with a median survival of only 12–16 months despite aggressive therapies including surgery, radiation and chemotherapy using alkylating agents such as temozolomide (TMZ) [1, 2]. GBM and many other cancers undergo metabolic adaptation and reprogramming, favoring a shift towards anabolic metabolism, with elevated aerobic glycolysis and oxidative phosphorylation (OXPHOS). This metabolic shift confers several tumorigenic advantages to GBMs, namely provision of biomass/macromolecules for growth, as well as production of excess lactate that favors tumor cell invasion and adaptation to unfavorable microenvironmental conditions like hypoxia or chemotherapy. In GBM, alterations to metabolic enzyme expression patterns and activity are highly recurrent [3, 4]. In low-grade gliomas and secondary GBM, highly recurrent mutations are observed in a key metabolic enzyme, *isocitrate dehydrogenase-1* (*IDH1*) [5, 6]. These hot-spot mutations result in a single amino acid substitution from arginine to histidine at position 132 (R132H), which leads to neomorphic activity of IDH1, and production of the onco-metabolite 2-hydroxyglutaric acid (2-HG), which in turn results in aberrant DNA methylation [7–10].

Another key driver of tumor metabolism is Hexokinase 2 (HK2), an enzyme that converts glucose to glucose-6-phosphate. This is an essential rate-limiting step for both glycolysis and ultimately oxidative phosphorylation [4, 11]. The link between HK2 overexpression and highly glycolytic malignant tumors was first demonstrated by mRNA analysis indicating that HK2 expression was increased by ~100-fold in tumor cells relative to normal cells [12, 13]. HK2 has a critical role in cancer progression, and its overexpression is associated with poor prognosis in many cancer types [14–17]. In GBMs, we have previously shown that up-regulation and over-expression of HK2 plays a key role in regulating a metabolic switch referred to as the “Warburg effect” [18]. In highly glycolytic GBM cell lines, mitochondrial-bound HK2 inhibition by constitutive HK2 knockdown was shown to inhibit aerobic glycolysis, leading to an increase in normal oxidative respiration, and a decrease in lactate production and induction of apoptosis, especially under hypoxic conditions [18]. In addition to regulating glycolysis, HK2 prevents Cytochrome-c release from mitochondria, and thereby inhibits apoptosis [19–22]. Multiple groups have investigated the possibility of developing and testing inhibitors of glycolysis as novel therapeutics, either singularly or in combination with other anti-cancer agents such as radiotherapy or chemotherapy. There are several lines of evidence that show increased efficacy of glycolysis inhibitors (such as 3- bromopyruvate (3-BRP) and 2 deoxyglucose (2DG)) when used alongside radiation and chemotherapy, and this likely works through promotion of apoptotic cell death [23, 24]. Combination of 2DG with standard chemotherapeutic agents adriamycin or paclitaxel results in an enhanced therapeutic activity in preclinical models of osteosarcoma and non-small-cell lung cancer [25]. Previous work from our group has shown that loss of HK2 diminishes GBM growth, and that HK2 is both tumor specific and not significantly expressed in normal adult human or murine brain [18, 26]. In this study we build on our prior work to show that HK2, but not the other brain-specific isoforms of hexokinase, HK1 and HK3, drives tumor metabolism in GBM. Moreover, we assess the effect of HK2 loss in combination with TMZ and/or radiation, and also further interrogate the mechanisms by which loss of HK2 results in radio-chemosensitivity.

## RESULTS

### Correlation of HK2 expression with glioma tumor grade and molecular signatures

**Figure 1:**
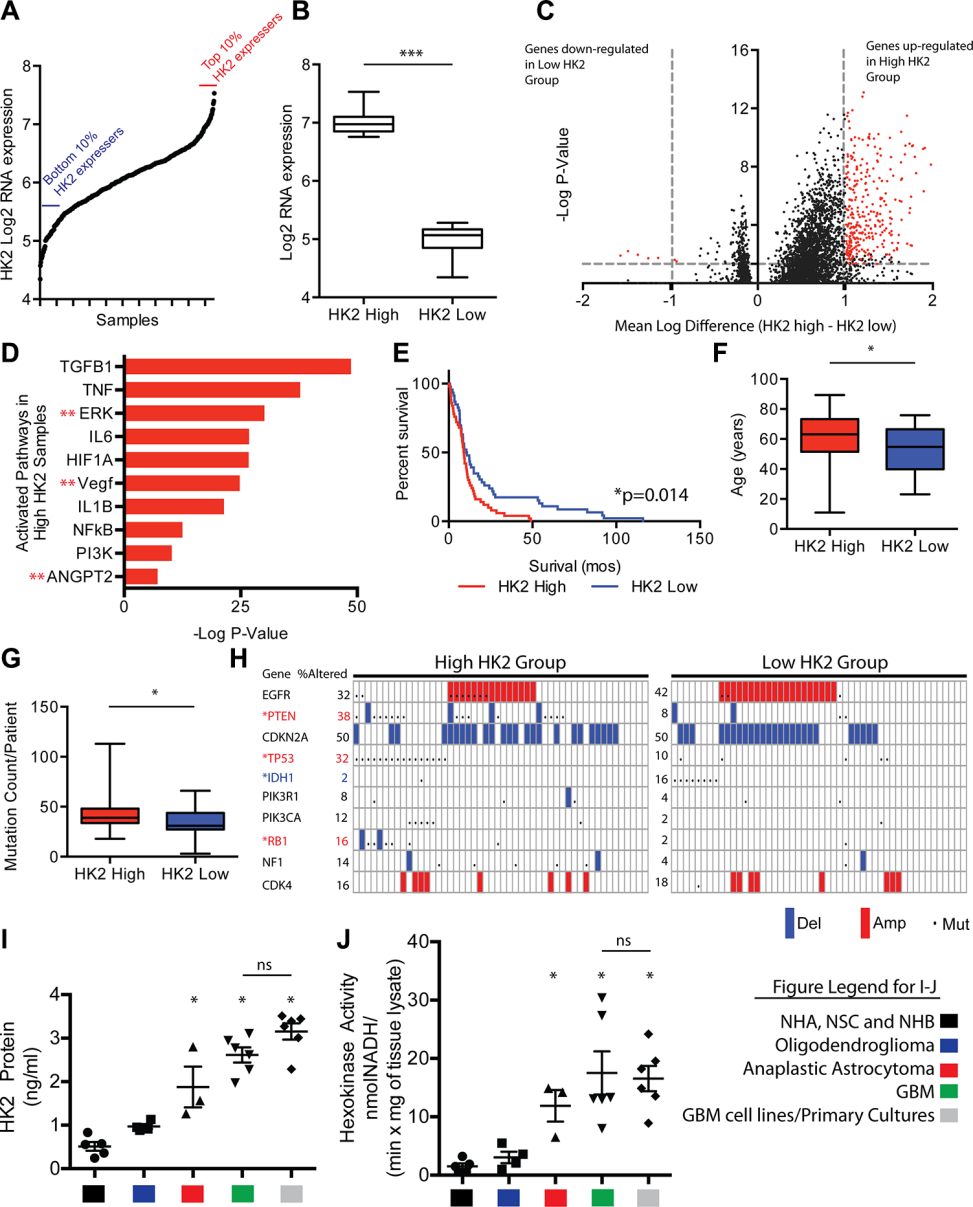
HK2 high expression is associated with aggressive clinical and molecular GBM features (**A**) HK2 gene expression distribution in over 500 TCGA GBM samples. The top 10% (*n* = 50) and bottom 10% (*n* = 50) of HK2 expressers were selected for further evaluation. (**B**) HK2 fold change at the RNA level in our two cohorts selected from A. (**C**) Volcano plot identifying differentially expressed genes between high and low HK2 GBM expressing patients. (**D**) Ingenuity pathway analysis of differentially expressed genes identifies several oncogenic pathways between high and low HK2 expressing patients. (**E**) Survival difference among top 10% and bottom 10% HK2 expressers selected from A. (**F**) Comparison of age between high and low HK2 expressers. (**G**) Comparison of average mutation count per patient between high and low HK2 patients. (**H**) Alterations in hallmark GBM genes between high and low HK2 groups. *Red stars indicate significantly mutated in high the HK2 cohort and *blue stars (IDH1) indicates mutation enrichment in the low HK2 cohort. (**I**) Hexokinase 2 specific ELISA measuring HK2 protein from various groups. Control group (normal brain samples (*n* = 3), NHA, NSC), oligodendroglioma (IDH1 mutant, grade II, *n* = 5), anaplastic astrocytoma (IDH1 mutant grade III, *n* = 3), GBM (grade IV, *n* = 6) and GBM cell lines and primary cultures (U87, GS2, GSC 8–18, GSC 7–2, TWH-1, TWH-2). (**J**) Hexokinase activity assay measured at 60 mins on samples from Figure 1I.

Using The Cancer Genome Atlas (TCGA) data set of 529 samples, we selected the top and bottom 10% of HK2 expressing patient samples (*n* = 50 per group) at the RNA level to identify molecular or clinically aggressive features of GBM associated with HK2 (Figure 1A). We observed a 4.5 fold change in HK2 expression between the two groups (Figure 1B, ***p < 0.001). We next compared differential transcript levels between the two groups using normalized level 3 U133A microarray data from the TCGA (Figure 1C). We identified 314 genes that were up-regulated, and 7 down-regulated genes within the high HK2 expressing group when compared to the low HK2 expressing group, using a minimum 2-fold difference and a Bonferonni corrected *p-value* of < 0.05 (Figure 1C). To identify molecular signatures of transcripts associated with high HK2 expression, we performed pathway analysis. As represented in Figure 1D, we observed significant enrichment in several oncogenic signaling pathways associated with HK2, including TGFB1, ERK, NFkB, PI3K and ANGPT2 signaling. Interestingly, we only identified 7 genes that were down-regulated when comparing high versus low HK2 expressing patients, and these genes were involved in both neuro-protection from Alzheimers signaling and TLX signaling (Supplementary Figure S1A). We next compared our high and low HK2 cohorts for additional molecular and clinical differences. Clinically, the high HK2 expressing cohort had decreased overall survival (Figure 1E, *p < 0.05). We observed no difference in O-6-Methylguanine-DNA Methyltransferase (MGMT) promoter methylation between the two groups, a known predictor of response to TMZ therapy in GBM (Supplementary Figure S1B). High HK2 expressing patients were also significantly older, and carried a higher mutation count per patient compared to low HK2 expressing patients (Figures 1F–1G). The high HK2 expressing cohort were significantly enriched for mutations or copy number alterations in PTEN, TP53 and RB1, while low HK2 expressing TCGA patient samples were enriched in IDH1 mutations (Figure 1H).

We next explored differential expression of all HK isoforms (HK1, HK2 and HK3) in all GBM patients using TCGA, comparing normal brain to the various molecular subtypes of GBM [27, 28]. Compared to normal brain, HK1 was significantly down regulated in all GBM subtypes by an average of 4 fold (Supplementary Figure S1C, ***p < 0.001), while HK2 was significantly up-regulated by an average of 5 fold in all subtypes of GBM (Supplementary Figure S1D, ***p < 0.001). HK3 showed a significant but modest increase of 1.3 fold in only the mesenchymal subtype of GBM compared to normal brain (Supplementary Figure S1E, **p < 0.01). Analysis of an additional dataset, the REpository for Molecular BRAin Neoplasia DaTa (REMBRANDT) [29], showed that increased expression of HK2 was associated with overall poorer survival in GBM patients (Supplementary Figure S1F, *p = 0.05). In contrast, increased expression of HK3 did not correlate with poorer overall survival, nor did reduced HK1 expression (Supplementary Figure S1G–S1H).

Surgically resected patient operative samples demonstrated a significant increase in HK2 expression (5 fold higher, *p < 0.05) when measured by ELISA assay in GBM patients when compared to normal controls [normal human brain (NHB)], normal human astrocytes (NHA) and neural stem cells (NSC)], and was 2.7 fold higher in HK2 (*p < 0.05) when compared with lower-grade oligodendrogliomas (grade II, IDH1 mutant) (Figure 1I). HK2 protein expression measured in 2 GBM cell lines (U87 and GS2) grown in 10% FBS and 4 primary GBM cultures grown in glioma stem cell media (GSC 8–18, GSC 7–2, TWH1, TWH2) exhibited on average 1.2 fold higher HK2 protein expression, although this was non-significant (Figure 1I, ns; *p* = 0.071). Similar to our HK2 protein results, primary GBM operative samples and GBM cultures had significantly higher hexokinase activity compared to normal controls (11 fold higher, (*p < 0.05)) and 6 fold higher (*p < 0.05) when compared to grade II gliomas (IDH1 mutant oligodendrogliomas) (Figure 1J, *p <* 0.05). Anaplastic astrocytoma (grade III) IDH1 mutant samples were significantly higher for HK2 expression and HK2 activity when compared to normal controls and grade II gliomas (*p < 0.05), but not significantly (ns) different from GBM and GBM cultures (Figure 1I–1J).

### HK2 but not HK1 or HK3 normalizes tumor metabolism and impacts both cell growth and survival

**Figure 2:**
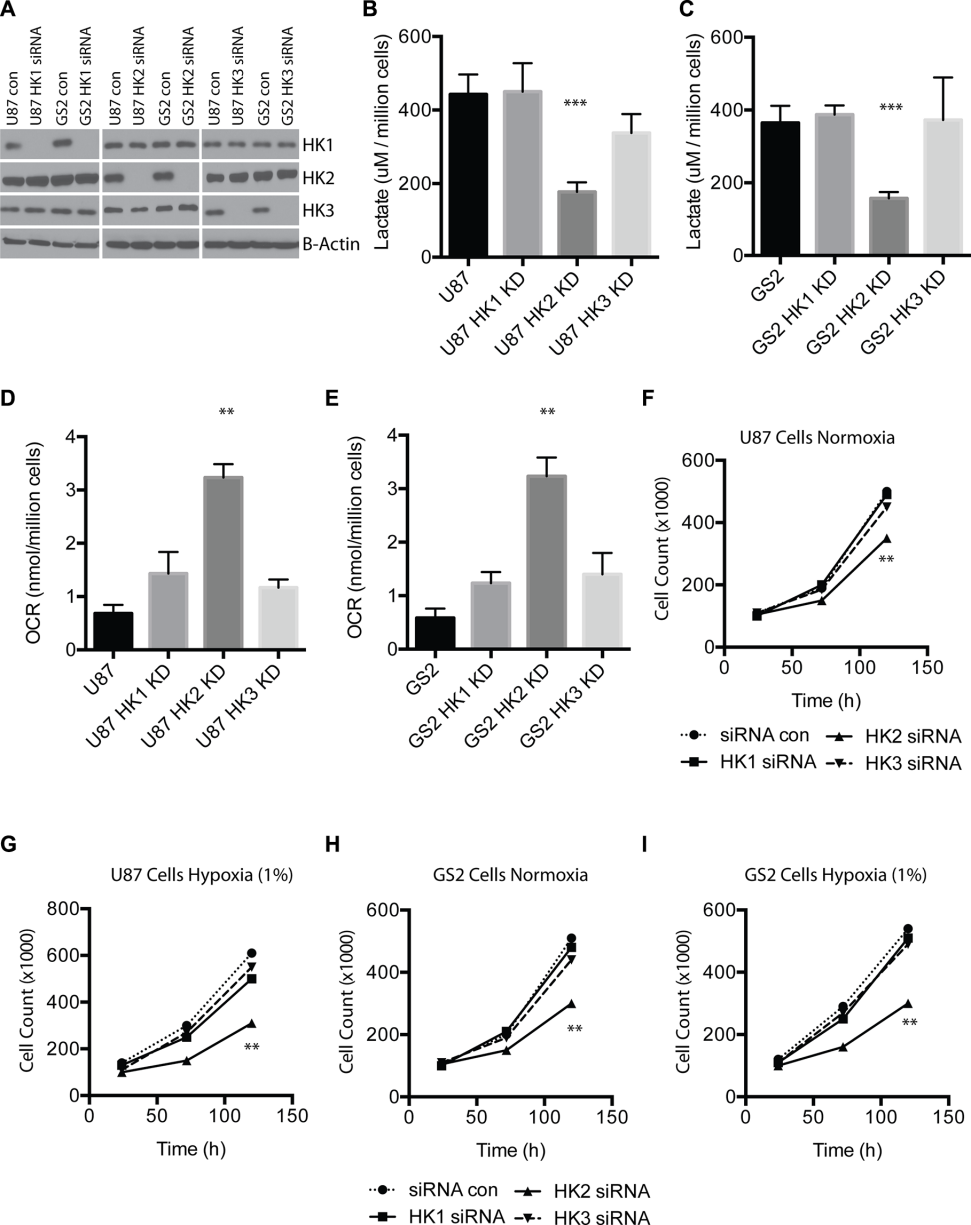
HK2 but not HK1 or HK3 impact tumor metabolism and cell growth (**A**) U87 and GS2 cells were transfected with control or pooled siRNAs targeting HK1, HK2, or HK3 at 10 nmol. Western blotting was performed 72 h later to confirm specific HK loss and no off-target effect on other HKs. (**B**) Lactate measurements for U87 (scrambled siRNA; Control) and U87 transfected with siRNA targeting HK1-3 after 72 h of siRNA treatment. (**C**) Lactate measurements for GS2 (scrambled siRNA, Control) and GS2 transfected with siRNA targeting HK1-3 after 72 h of siRNA treatment. (**D**) Oxygen consumption by U87 (scrambled siRNA, Control) and U87 transfected with siRNA targeting HK1-3 after 72 h of siRNA treatment. (**E**) Oxygen consumption by GS2 (scrambled siRNA, Control) and GS2 transfected with siRNA targeting HK1-3 after 72 h of siRNA treatment. (**F**–**I**) Cell growth assay for U87 and GS2 cells in normoxia or hypoxia (1%) treated with control siRNA or HK-specific siRNA. All experiments were performed in triplicate and * indicates *p* < 0.05; **, *p* < 0.01; ***, *p* < 0.001.

Western blotting of U87 and GS2 cell line lysates transfected with pooled siRNA targeting HK1, HK2, HK3 and scrambled siRNA confirmed efficient knockdown of all three HK isoforms at the protein level. We observed no off-target effects of the HK specific siRNA pools on other hexokinase isoforms, validating the specificity of the siRNA (Figure 2A). Loss of HK2 but not HK1 or HK3 reduced lactate production (Figure 2B–2C, ***p < 0.0001), and loss of HK2 resulted in the largest increase in oxygen consumption in both U87 and GS2 cells (Figure 2D–2E, **p < 0.001). HK2 was also the only isoform that exhibited reduced cell growth as measured by direct cell counts over 5 days in both cell types, and this effect was further increased when cells were grown in hypoxia (1% O_2_) (Figure 2F–2I**p < 0.001).

### Generation of inducible HK2 knockdown GBM cells and its impact on tumor metabolism

We next generated and characterized doxycycline (dox) inducible HK2 knockdown (HK2 KD) cell lines using two different HK2 shRNA hairpin sequences in an established GBM cell line (U87) and GS2 cells. Western blot analysis of doxycycline-inducible HK2 KD confirmed significant knockdown (> 90%) of HK2 after 72 hours in U87 and GS2 cells (Supplementary Figure S2A–S2D). No off-target effects on HK1 or HK3 were observed, demonstrating the specificity of HK2 shRNA sequences (Supplementary Figure S2). Inducible U87 HK2 KD cells resulted in significantly greater oxygen consumption rate and decreased lactate production (Supplementary Figure S2E–2H), similar to our transient siRNA observations.

### Loss of HK2 increases survival in GBM xenografts and is tumor stage-dependent

**Figure 3:**
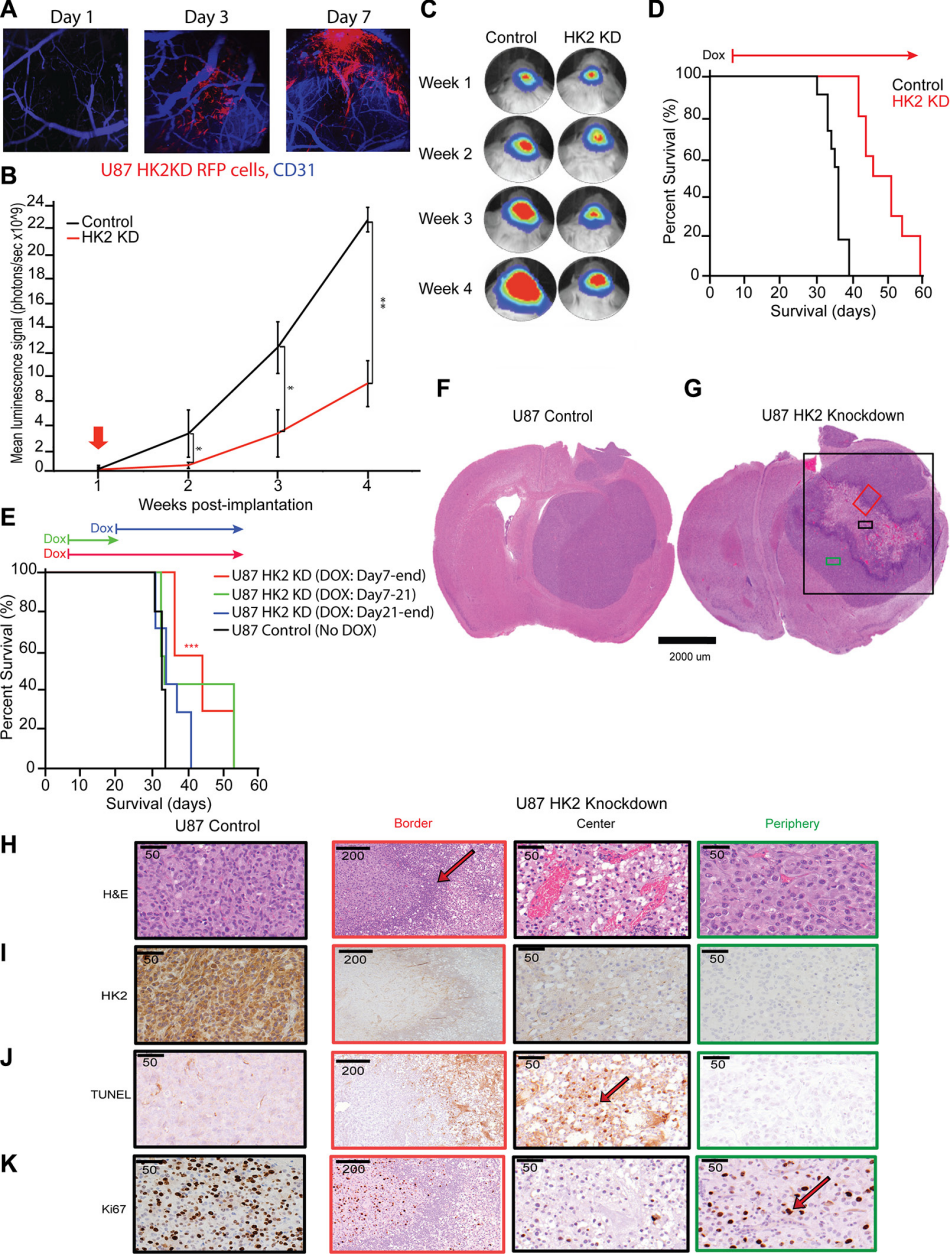
Loss of HK2 increases survival in GBM xenografts and is stage dependent (**A**) *In vivo* 2 Photon Laser Microscopy imaging for U87 control and U87 HK2 KD mice with xenograft models generated in an intracranial window chamber. Representative images from Day 1, Day 3 and Day 7 post-dox administration. Blue is CD31 staining for murine brain vessels, and red is U87 HK2 shRNA cells. By Day 3 post-dox, red cells can be visualized in U87 HK2 KD group confirming that the transgene is on (HK2 shRNA), while the red signal is absence in the control group. (**B**) Bioluminescence imaging (BLI) of xenografted U87 HK2 KD (dox treated) (*n* = 10), and U87 control (No dox) (*n* = 10). (**C**) Quantification of BLI signal of tumor region from week 1 to 4 (A) with representative heat map image of mice with U87 tumors +/- HK2 KD. Doxycycline (dox) was administered from day 7 to the end of the experiment. (**D**) Kaplan–Meier survival curve showing mice with HK2 KD (dox treated) survived significantly longer compared to control mice. (**E**) Kaplan-Meier survival curve of mice with intracranial U87 HK2 shRNA cells treated with dox at different time points. The survival of mice with U87 tumors treated with dox from day 7-end (*n* = 7) was significantly improved from those treated with dox from day 7–21 (*n* = 7), day 21-end (*n* = 7) or control no dox (*n* = 5). (**F**–**G**) Representative H&E brain sections from U87 control tumors and U87 HK2 KD (plus dox) tumors. U87 HK2 KD tumor sections were divided into three regions of center (black box), periphery (green box) and border (red box). (**H**–**K**) Immunohistochemical (IHC) analysis of control tumors and HK2 KD tumors (dox treated) at the center, periphery and border for HK2, ki67, TUNEL and HIF1a.

Having established the transient and stable effect of HK2 loss *in vitro*, we next explored the role of HK2 KD cells *in vivo*. Monitoring of mice injected with U87 HK2 KD cells using intracranial window based 2PLM imaging [30, 31] demonstrated successful expression of RFP-HK2-shRNA after 3 days of continual dox administration, confirming dox penetration of the blood brain barrier and activation of our HK2 shRNA transgene in an inducible manner (Figure 3A). The secondary reporter tag luciferase was used for Bioluminescence Imaging (BLI) to monitor tumor growth. The growth of U87 HK2 KD tumors was significantly decreased relative to controls from week 2 onwards (Figure 3B, 3C). The median survival of mice injected with HK2 KD glioma cells was significantly increased from 36 days to 48 days when compared to mice without HK2 knockdown (Figure 3D, ***p < 0.001). Our group has previously shown that stable knockdown of HK2 increases survival in GBM, but the temporal effect of HK2 knockdown or duration required for survival benefit has never been assessed. To address this, analysis of survival data between 4 differently timed dox administration groups revealed that dox had the most effect when administered early (day 7) and continuously throughout the experiment (group 3) 45 days vs 33 days. (Figure 3E, ***p = 0.001). Shorter dox treatments and those started after 21 days did not result in a significant survival benefit compared to the control groups (Figure 3E).

### Histopathological characterization of HK2 depleted tumors

Histopathological analysis demonstrated that U87 control tumors grew comparatively well-circumscribed, as previously reported [32], with no evidence of necrosis or invasion (Figure 3F). Comparatively, HK2 KD tumors demonstrated distinctive histological regions, including a necrotic center surrounded by proliferative tumor cells in the periphery, and a border zone containing predominantly neutrophils (Figure 3G and Supplementary Figure S3A). For further analysis, we divided the HK2 KD tumors based on these three distinct cellular phenotypes into central, border and peripheral regions based on H and E staining (Figure 3H). HK2 KD tumors showed significantly reduced HK2 staining (Figure 3I). TUNEL staining was used in order to measure apoptosis, and was only observed in U87 HK2 KD tumors in the central necrotic region (Figure 3J and Supplementary Figure S4A, ***p < 0.001), with minimal non-significant TUNEL staining in U87 control tumors. Cleaved Caspase 3, an additional marker of apoptosis, was also observed at the areas bordering the inflammatory cells in U87 HK2 KD tumor sections, and was absent in U87 control tumors (Supplementary Figure S3B). We observed a 55% reduction in proliferation in U87 HK2 KD xenografts as quantified by Ki67 (Figure 3K and Supplementary Figure S4B, ***p < 0.001).

### Loss of HK2 alters tumor vasculature

**Figure 4:**
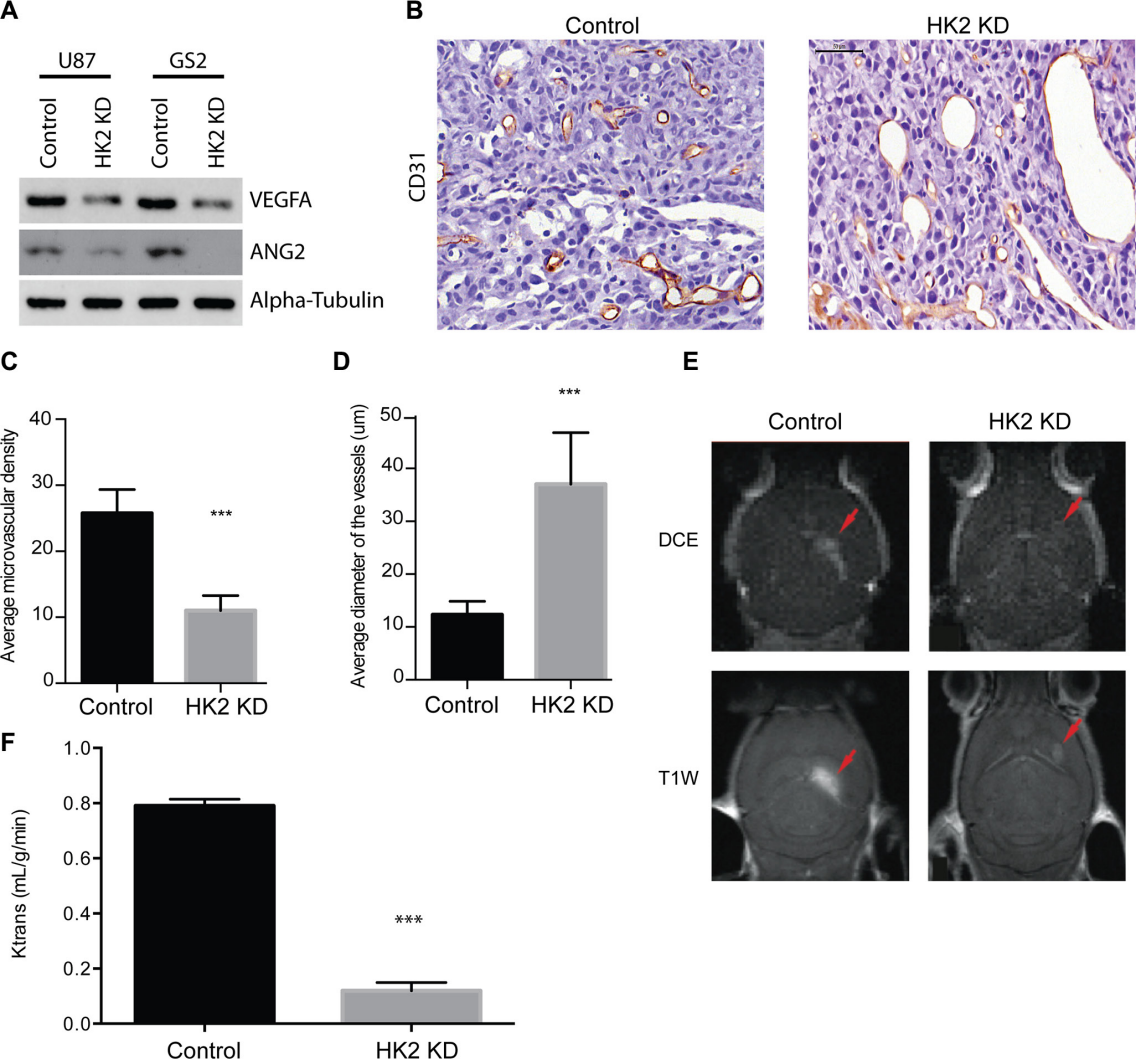
HK2 loss diminishes tumor vasculature in a GBM xenograft model (**A**) Western blotting of ANGPT2(ANG2) and VEGFA in U87 and GS2 cells with and without HK2 knockdown. (**B**) Immunohistochemical (IHC) staining of CD31 in U87 control and the periphery region of U87 HK2 KD tumor sections. HK2 knockdown was accomplished by 48 h of doxycycline administration to tumor cells. (**C**) Quantifications of microvascular density (MVD) of control tumors versus HK2 KD tumors (with dox). (**D**) Quantifications of microvascular diameter of control tumors versus HK2 KD tumors (with dox). (**E**) Mice with U87 tumors +/- HK2 KD were imaged by MRI three weeks post-implantation. Representative dynamic contrast-enhanced (DCE) MR image (top frame) and T1-weighted (T1w) MR images (bottom frame) are shown. (**F**) Vascular permeability was measured using K_trans_ values calculated from brain scans. Scale bar denotes 50 um.

Microscopic analysis of HK2 KD tumors versus control identified altered vasculature. We hypothesized that the extended survival observed in our mice by inhibiting tumor glycolysis through HK2 loss may be a consequence of altered tumor neo-vascularization. This is further supported by the observations that high HK2 expressing GBM patients have significant enrichment in both VEGF and ANG signaling pathways that are essential for angiogenesis (Figure 1D). To account for these vascular alterations, we examined expression of two key groups of angiogenic factors involved in GBM neo-vascularization: Vascular endothelial growth factor A (VEGFA) and angiopoietin 2 (ANG2)/angiopoietin 1 (ANG1), in response to a decrease in HK2. Loss of HK2 resulted in a significant reduction in both VEGFA and ANG2 in U87 and GS2 cell lines (Figure 4A). Tumor vessels were visualized using CD31 staining, which reflects endothelial cell (EC) staining. Overall, CD31 staining was significantly decreased in HK2 KD tumors, particularly in the peripheral region away from the necrotic core when compared to control tumors (Figure 4B). Associated with a decrease in EC staining, there was a 55% reduction in microvascular density (MVD) (Figure 4C, ***p < 0.001) and a 61% increase in microvascular diameter throughout the HK2 KD tumors relative to control tumors (Figure 4D, ***p < 0.001), supporting a decreased flow and perfusion as seen in MRI. Histopathological analysis of the tumor vasculature, mice bearing intracranial GBM xenografts for both control and HK2 KD underwent dynamic contrast-enhanced (DCE) magnetic resonance imaging (MRI), a technique we previously utilized to measure K_trans_ as a marker for vessel perfusion and permeability [33]. Mice with HK2 KD tumors showed smaller tumors compared to control, confirming a reduction in tumor size, using both DCE-MR images (Figure 4E, top panel) and T1-weighted with contrast (T1w) MR images (Figure 4E, bottom panel). In addition, vessel perfusion/permeability as measured by K_trans_ was significantly reduced in HK2 KD tumors compared to control (Figure 4F, ***p < 0.001).

### Loss of HK2 sensitizes tumors to chemotherapy and radiation *in vitro* and *in vivo*

**Figure 5:**
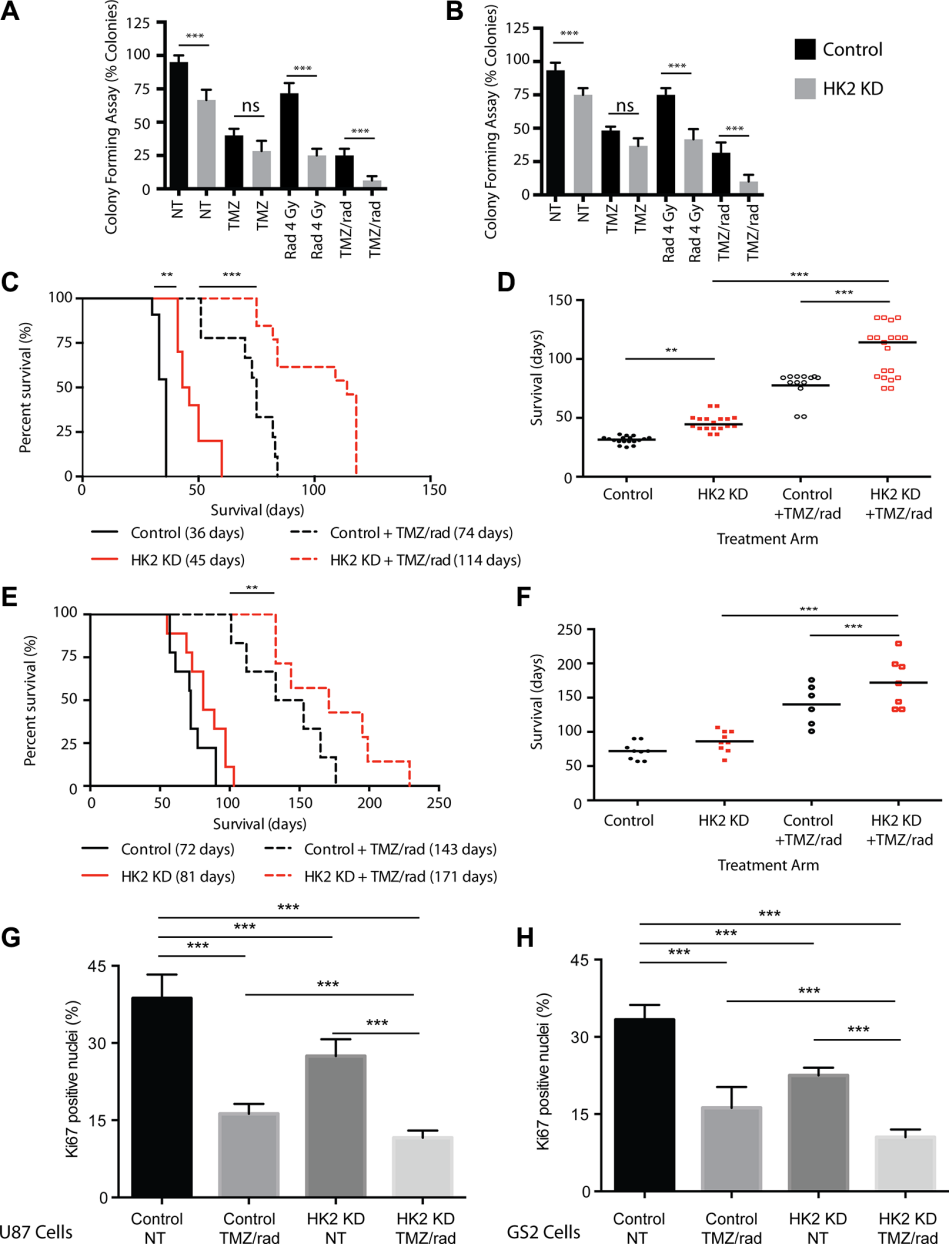
Loss of HK2 sensitizes tumors to chemotherapy and radiation (rad) (**A**) Colony forming Assay: U87 cells were treated with dox or no dox to induce HK2 loss. 72 h post dox treatment, cells were treated with vehicle (0.1% DMSO), TMZ (100 uM), 4 Gy Rad or combined treatment (TMZ/radiation). Colonies were scored on Day 14 and data represented as % colony forming units relative to non-treated control cells. (**B**) Colony forming Assay for GS2 cells, see A for details. (**C**) Kaplan–Meier survival curve showing percent survival of mice bearing U87 tumors randomized to with dox (HK2 KD) (*n* = 11) or No dox (control) (*n* = 10) and treated with combined TMZ/radiation (*n* = 8 No dox and *n* = 12 (with dox). Median survival is in brackets. (**D**) Rain plot demonstrating median survival in U87 xenografts from A each dot represents a mouse on the rain plot. (**E**) Kaplan–Meier survival curve showing percent survival of mice bearing GS2 tumors randomized to with dox (HK2 KD) (*n* = 9) or No dox (control) (*n* = 9) and treated with combined radiation and TMZ (*n* = 7 No dox and *n* = 7 With dox). Median survival is in brackets. (**F**) Rain plot demonstrating median survival in GS2 xenografts from C each dot represents a mouse on the rain plot. (**G**) Ki67 quantifications for tumor sections in A. 5 mice per arm were stained and 20 fields per mouse were used to quantify fraction Ki67 positive cells. Bars are normalized to U87 control. Bars represent relative mean values ± SD, and asterisks denote a significant difference. (**H**) Ki67 quantifications from tumor sections. 5 mice per arm were stained and 20 fields per mouse were used to quantify fraction Ki67 positive cells. Bars are normalized to GS2 control. Bars represent relative mean values ± SD, and asterisks denote a significant difference.

**Figure 6:**
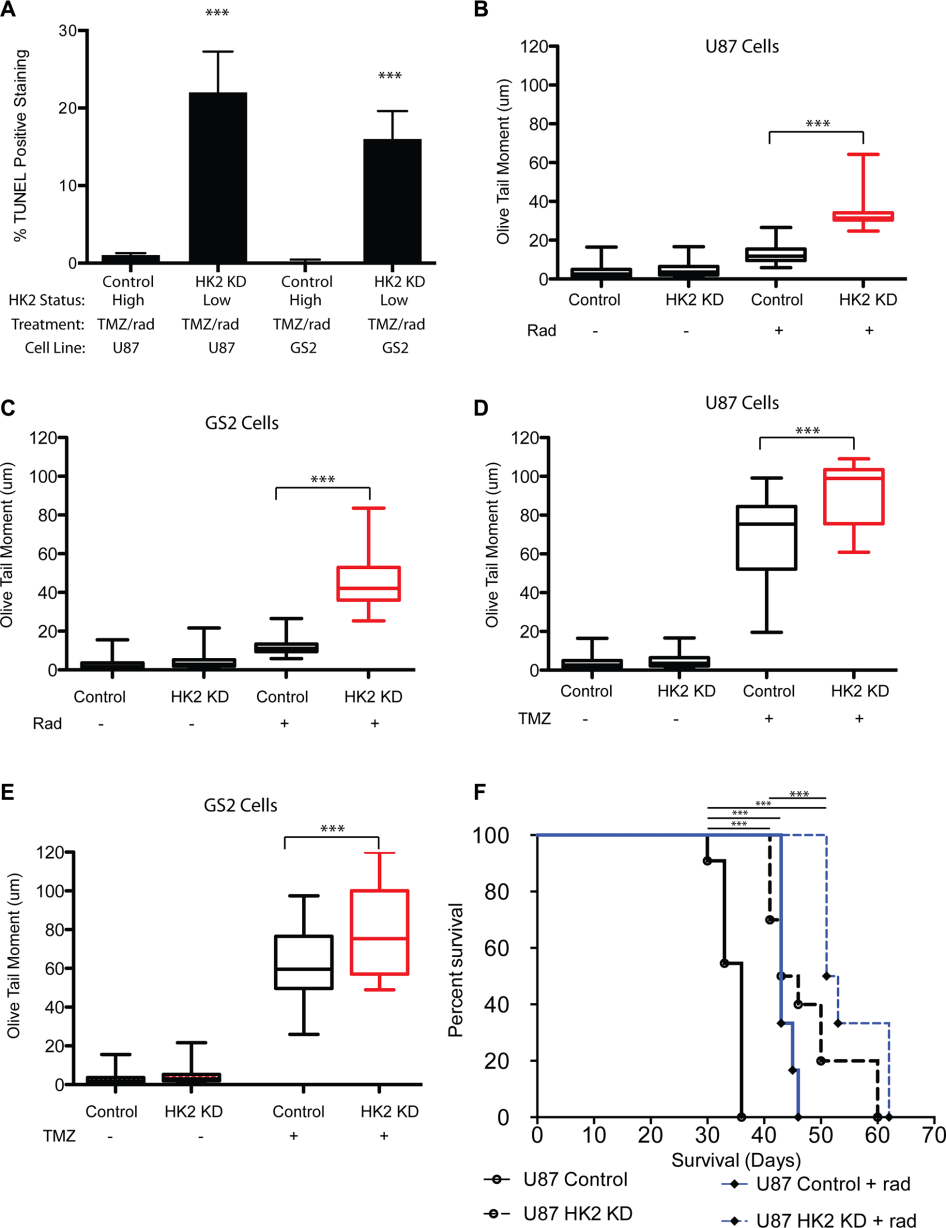
Loss of HK2 increases DNA-damage (**A**) TUNEL quantifications from tumor sections in Figure 4. 5 mice per arm were stained and 20 fields per mouse were used to quantify fraction TUNEL positive cells. (**B**–**C**) Comet tail assay measuring DNA damage on day 2 in U87 and GS2 cells with or without HK2 (plus or minus doxycycline) treated with TMZ alone (100 uM). (**D**–**E**) Comet tail assay measuring DNA damage on day 2 in U87 and GS2 cells with or without HK2 (plus or minus doxycycline) treated with radiation (4 Gy). (**F**) *In vivo* orthotropic xenograft model of U87 cells and U87 cells plus dox (HK2 KD), treated with or without radiation (rad).

Using a colony-forming assay to measure clonogenic potential and cell viability, radiation significantly reduced colony-forming potential in HK2 KD cells compared to control cells (Figure 5A–5B, ***p < 0.001). We observed no significant difference (ns) under TMZ treatment conditions between HK2 KD and control cells. However, combined TMZ/radiation had the largest effect, by significantly reducing colony-forming units in U87 and GS2 HK2 KD cells compared to controls (Figure 5A–5B, ***p < 0.001). We next tested loss of HK2 *in vivo* in combination with a human equivalent of TMZ/radiation treatment on GBM mice xenograft models. [1, 34]. Mice were randomized into 4 groups: HK2 KD (dox treated), HK2 KD with concomitant TMZ/radiation, control (no treatment), and control with concomitant TMZ/radiation. Log-Rank survival analysis demonstrated that median survival of mice with HK2 KD was significantly increased to 44.5 days versus 36 days in the control group (Figure 5C–5D,**p < 0.01). Mice treated with concurrent TMZ/radiation and HK2 KD had the greatest survival benefit, 114 days versus 74 days (a 154% increase) compared to control treated xenografts (Figure 5C–5D, ***p < 0.001). Similarly, loss of HK2 in an additional orthotopic xenograft model of GS2 cells led to increased survival approaching significance (Figure 5E–5F, *p* = 0.065). Under TMZ treatment conditions, GS2 HK2 KD xenografts had the greatest survival benefit when compared to GS2 control xenografts treated with TMZ/radiation, 180 vs 140 days respectively (Figure 5E–5F, **p < 0.01). Loss of HK2 in both U87 and GS2 xenograft models led to significantly reduced Ki67 staining, which was lowered further by TMZ/radiation treatment (Figure 5G–5H, ***p < 0.001). Doxycycline induced HK2-loss was confirmed in both U87 and GS2 xenograft models by IHC (Supplementary Figure S4C–S4D). Apoptosis was significantly increased in U87 and GS2 HK2 knockdown TMZ/radiation treated xenograft models as determined by TUNEL staining when compared to control xenografts treated with TMZ/radiation (Figure 6A, ***p < 0.001) and Supplementary Figure S4C–S4D).

### Loss of HK2 induces DNA damage

We evaluated the impact of radiation and TMZ alone to determine whether HK2 loss sensitized cells equally or preferentially to TMZ or radiation with respect to DNA damage and cell viability. Treatment with radiation (4 Gy) significantly increased DNA damage as evaluated by the comet tail assay in HK2 KD cells, but not control cells (Figure 6B–6C, ***p < 0.001). Treatment with TMZ increased DNA damage in control cells, however HK2 KD cells had a further significant increase in DNA damage compared to control cells (Figure 6D–6E, ***p < 0.001). Our *in vitro* DNA damage results suggested that radiation selectively targeted tumor cells with HK2 loss, whereas TMZ induced DNA damage showed only a modest increase in HK2 KD cells compared to TMZ treated control cells. Based on these observations, we tested whether radiation alone would be sufficient to extend xenograft survival *in vivo*. U87 orthotopic xenograft models treated with radiation alongside doxycycline induced HK2 KD had the greatest survival compared to control groups (Figure 6F, ***p < 0.001).

### Loss of HK2 in a low passage primary GBM culture inhibits tumor metabolism and sensitizes GBM cells to TMZ/Radiation *in vitro*

**Figure 7:**
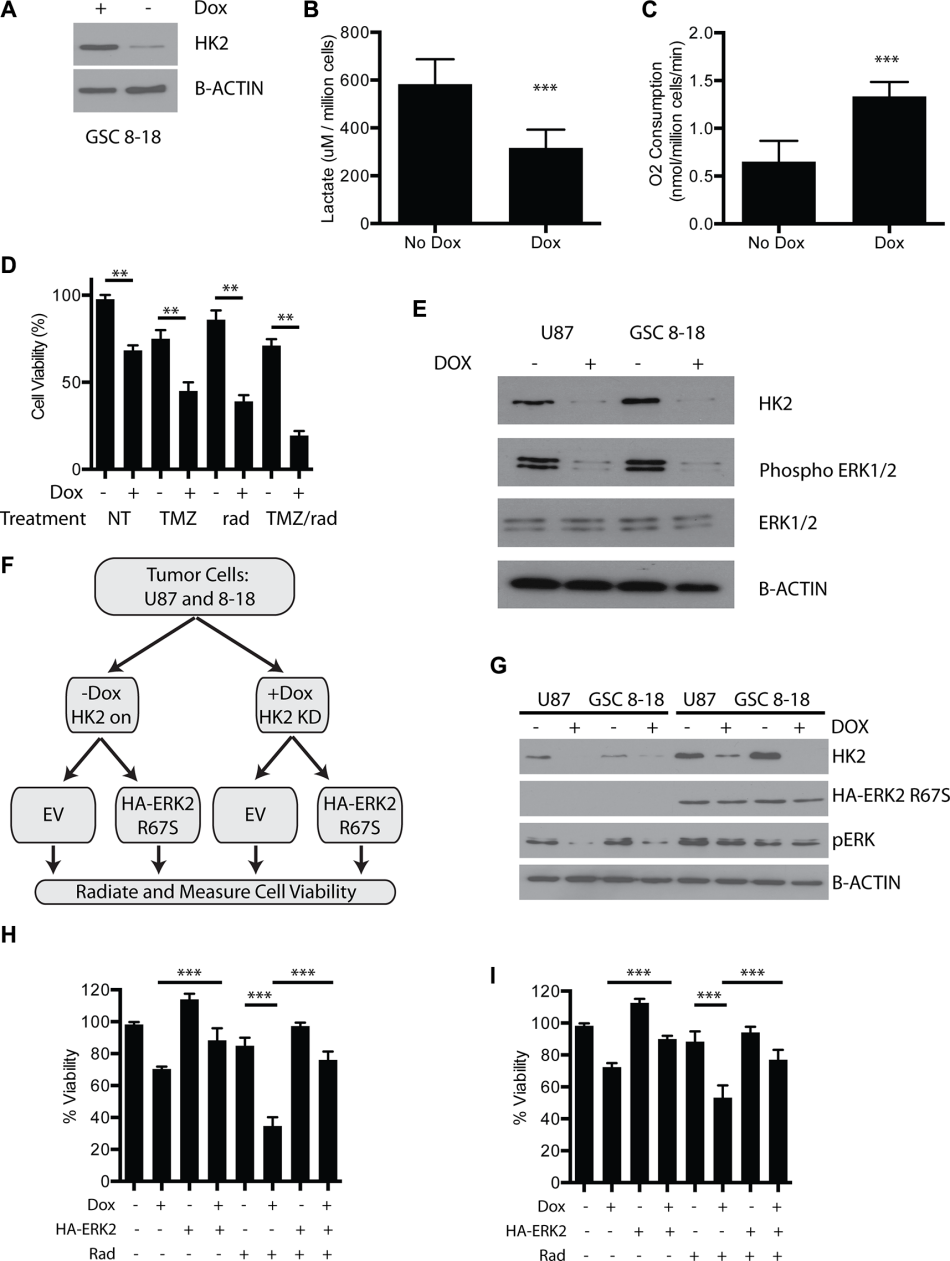
Loss of HK2 reduces ERK1/2 and promotes radiosensitivity (**A**) Immunoblot of HK2 in GSC 8–18 cells treated with or without doxycycline. (**B**) Lactate production of control GSC 8–18 cells and GSC 8–18 cells with HK2 knockdown (plus dox). (**C**) Oxygen consumption of control GSC 8–18 and GSC 8–18 with HK2 knockdown (plus dox). O_2_ consumption was normalized per million cells. (**D**) Viability of GSC 8–18 cells measured by Almar blue viability dye at 72 h in response to presence or absence of HK2 treated with or without TMZ/rad. (**E**) Loss of HK2 leads to reduced phospho-ERK1/2 levels as measured by immunoblotting. (**F**) ERK2 rescue experiment outline. Cells were transfected with empty vector or HA tagged ERK2 (R67S). After 24 transfection, cells were then seeded in 96 well plates (5000 cells per well) and treated with or without radiation and measured using an Almar blue viability assay at 72 h. (**G**) Immunoblot confirming presence of constitutively active HA tagged ERK2 (R67S) and HK2 knockdown by doxycycline in U87 and GSC 8–18 cells. (**H**) Viability of U87 cells measured by Almar blue viability dye at 72 h in response to presence or absence of constitutively active ERK2 (R67S) when treated with 4 Gy radiation (rad). (**I**) Viability of GSC 8–18 cells measured by Almar blue viability dye at 72 h in response to presence or absence of constitutively ERK2 (R67S) when treated with 4 Gy radiation.

The impact of HK2 loss in a primary GBM culture from an operative sample (GSC 8–18) that is grown in defined serum free media (glioma stem cell media) was next evaluated to determine if we observed similar phenotypes in low passage cells. We established a doxycycline inducible HK2-shRNA construct in our GSC 8–18 culture. Administration of dox inhibited HK2 expression as measured by western blot by day 3 (Figure 7A). As observed in our other GBM tumor cells, loss of HK2 in GSC 8–18 cells lead to significant reduction in lactate levels and increased oxygen consumption (Figure 7B–7C, **p < 0.001). Loss of HK2 led to reduced viability (evaluated by Almar Blue) compared to control cells, and this reduction was further enhanced when cells were treated with 100 uM TMZ and 4 Gy radiation (Figure 7D, **p < 0.01). The combination of HK2 loss with combined TMZ/radiation led to the greatest reduction of cell viability compared to all other control and treated groups (Figure 7D, **p < 0.01).

### Loss of HK2 reduces ERK to promote radio-sensitivity

Inhibition of ERK1/2 signaling has been shown to promote radio-sensitivity in glioma cells [35–37]. Furthermore, ERK1/2 has been shown to foster glycolytic gene expression and promote the Warburg effect [38]. Moreover, ERK signaling can drive cell proliferation in several tumors by altering mitochondrial carbon flux [39]. We postulated that loss of HK2 and inhibition of aerobic glycolysis would directly attenuate ERK signaling in GBM, as we observed enrichment of ERK signaling in the high HK2 GBM group (Figure 1D). Loss of HK2 led to reduced activation of phospho-ERK1/2 in both U87 cells and our primary 8–18 culture (Figure 7E). To understand the significance of reduced ERK signaling, we performed a rescue experiment with a constitutively active HA tagged ERK2 (R67S) construct (Figure 7F–7G). In U87 cells, inhibition of HK2 led to reduced cell survival compared to control cells with 4 Gy radiation (Figure 7H, ***p < 0.001). Expression of HA-ERK2 (R67S) rescued the viability defect of HK2 KD cells treated without radiation, and more importantly rescued HK2-reduced viability under radiation conditions (Figure 7I, ***p < 0.001,). Similar results were observed in GSC 8–18 cells (Figure 7I, ***p < 0.001).

### HK2 loss sensitizes GBM cells to glutamine inhibition

**Figure 8:**
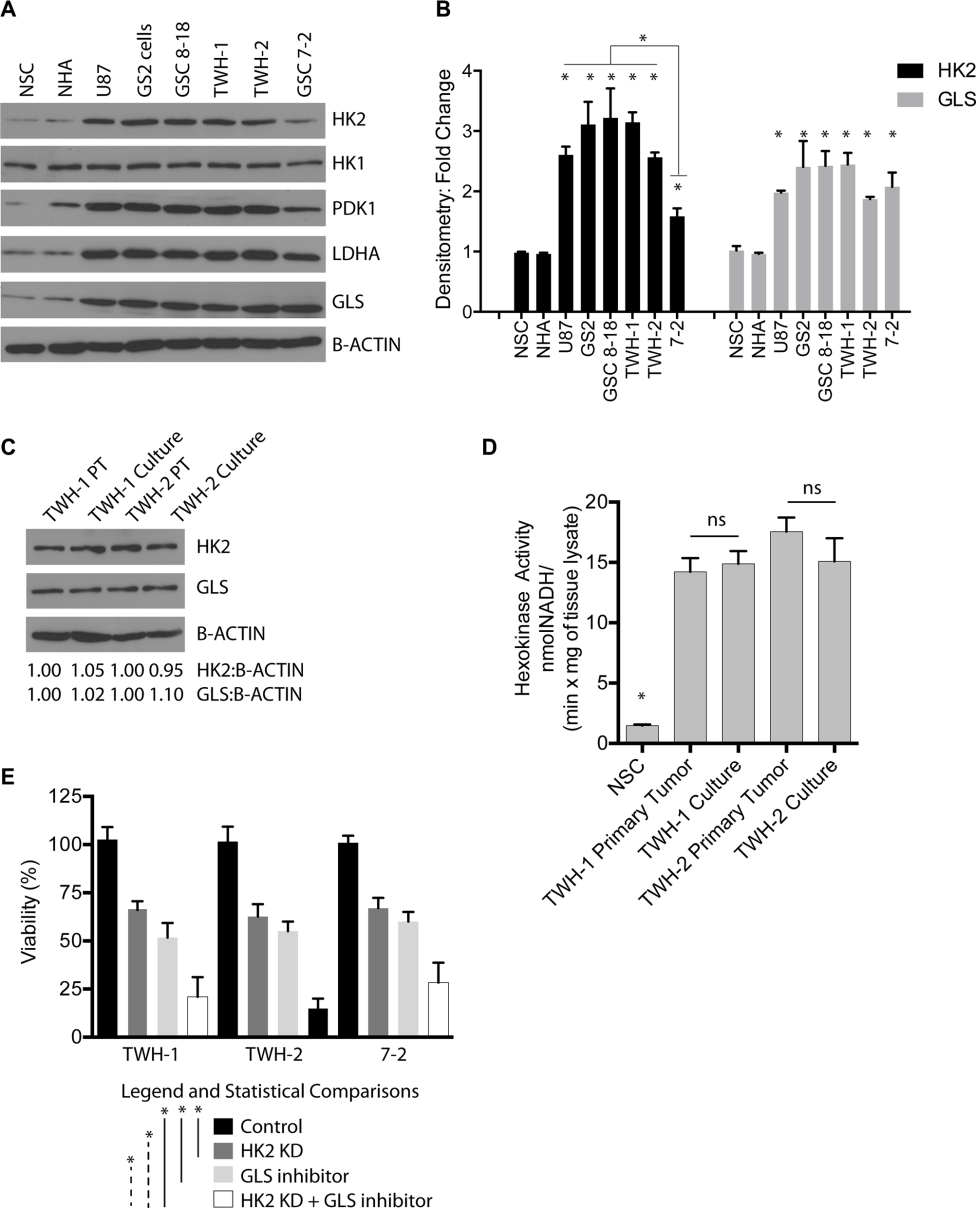
Loss of HK2 sensitizes cells to GLS inhibition (**A**) Western blot profiling of several GBM cell lines (U87 and GS2) and GBM primary cultures isolated and grown in glioma stem cell media. (**B**) Densitometric analysis of HK2 and GLS protein levels taken from three independent western blots. (**C**) Western blot comparing primary GBM operative sample to matched patient derived GBM culture at passage 5 for HK2 and GLS protein levels. (**D**) Hexokinase activity assay measured at 60 minutes comparing primary tumor to cell line derived from that tumor. (**E**) Almar blue Cell viability assay taken at 72 h on two primary GBM cell cultures treated with HK2 siRNA, 1 uM GLS inhibitor, HK2 siRNA with GLS inhibitor (compound 968, 1 uM) compared to control cells (treated with vehicle and siRNA control).

In addition to glucose, glutamine is a highly important and abundant amino acid found in the brain and in the media of cell cultures required for normal and cancer cell growth [40]. Glutamine is converted to glutamate by glutaminase (GLS), which can then be used for generation of several key metabolites and ATP production. We first profiled several GBM cell lines (U87 and GS2), GBM stem cells grown (GSC 8–18 and GSC 7–2) and two newly established GBM primary cultures growth in glioma stem cell conditions (TWH1 and TWH2) for several glycolysis markers, HK2, PDK1, LDHA, and GLS, the enzyme responsible for conversion of glutamine to glutamate (Figure 8A). Compared to normal human astrocytes and normal neural stem cells, all GBM cell lines/cultures were elevated for HK2, PDK1, LDHA, GLS but not HK1 (Figure 8A). Both HK2 and GLS were 2–3 fold higher with respect to protein expression as evaluated by densitometry analysis (Figure 8B, *p < 0.05), with LDHA and PDK1 following the same pattern. HK2 protein expression in TWH1 and TWH2 showed no significant difference in either HK2 protein expression or protein activity when compared to the primary tumor from which they were derived, supporting that cell culture conditions did not alter HK2 expression (Figure 8C–8D). Lastly, combined loss of HK2 by siRNA and a GLS inhibitor (Compound 968 at 1 uM) resulted in a significant reduction in cell viability versus HK2 knockdown or GLS inhibition alone (Figure 8E,*p < 0.05).

## DISCUSSION

The prognosis for GBM patients is extremely poor despite surgery and radio-chemotherapy, and targeting tumor metabolism may provide a novel avenue for inhibiting tumor metabolism. Refocusing research efforts with a focus towards altered tumor metabolism has established a link between cancer-specific metabolic anomalies and the principal hallmarks of cancer [41]. This link has led to novel interest in exploring therapeutic concepts aimed at inhibiting cancer-specific metabolic programs or drivers. To date, there has been no report of approaches involving pre-clinical combination of glycolysis inhibitors, such as HK2 inhibitors, alongside radiation plus TMZ in GBMs. Moreover, isozyme-specific inhibitors of HK2 have not yet been fully evaluated to precisely determine their therapeutic effect [42]. We demonstrate that the survival benefit provided by inhibition of HK2 is tumor growth stage-dependent. Disruption of tumor metabolism through HK2 inhibition must occur early in GBM treatment, and ideally HK2 inhibition would be prolonged and continuous, as this may lead to the greatest survival benefit.

HK2 loss, but not HK1 or HK3, had a significant effect on lactate production and oxygen consumption, and our clinical data suggests that higher HK2 expression correlated with poorer survival. Recently, using an HK2 conditional knockout mouse, a group showed that HK2 is required for tumor initiation and maintenance in mouse models of KRas-driven lung cancer [43], lending further evidence that HK2 is a key metabolic driver in cancer, and an attractive target in cancer biology. Since concomitant radio-chemotherapy is the standard of care in the clinic for GBM patients, new treatment strategies should be compared with the efficacy of that protocol. In this regard, our *in vivo* combinatorial therapy examined the additive effect of the triple regimen of HK2 knockdown with concomitant radio-chemotherapy. Our *in vivo* data showed that loss of HK2 resulted in a statistically significant increase in overall survival when compared to radiation and chemotherapy alone. Although HK2 elevation has been reported in several cancers compared to normal tissues, some normal tissues including muscle cells and normal cerebellum have HK2 protein [44]. Therefore there is a possibility that some toxicity with future HK2 inhibitors may exist if these agents are delivered systemically.

HK2 loss *in vivo* resulted in a significant decrease in both tumor vasculature and expression of angiogenic factors such as VEGFA and ANG2, which are key for regulating the hyper-proliferative vasculature in GBMs and triggering the recruitment of bone marrow derived cells in response to radiation, providing a possible escape mechanism for the tumor [31, 45]. These results provide sufficient basis to support further investigation into combining anti-angiogenic therapies with metabolic inhibitors.

Sensitization to radiation following HK2 loss may be due to downstream disruption of ERK 1/2 signaling, which has been shown to promote radio-resistance in glioma [35–37]. Loss of activation of these pathways through HK2 loss may partially explain the sensitivity to DNA damage. In support, a constitutively active ERK2 (R67S) construct was able to rescue the sensitivity to radiation in HK2 depleted cells. In addition to the Warburg effect, several studies have demonstrated that inhibition of glutaminolysis by inhibiting GLS can suppress cancer growth, including gliomas [46–48]. Our results support that dual targeting of glycolysis through HK2 knockdown and inhibition of glutaminolysis through GLS inhibition can be another effective treatment strategy.

Cancer cells, regardless of their genetic background, rely on changes in metabolism and reorganization of metabolic pathways to support growth and survival. Based on the results of this study, identification of specific HK2 inhibitors will be important as a therapeutic target.

## MATERIALS AND METHODS

### Cell lines and cell culture conditions

U87 cells (American Type Culture Collection (ATCC, Manassas, VA), and GS2 cells (a kind gift from Katrina Lamzus at the Hans-Dietrich Hermann Laboratory for Brain Tumor Biology), and normal human astrocytes were purchased from ABM (cat# T0281) were maintained in complete medium consisting of Dulbecco's Modified Eagle Medium (DMEM) supplemented with 10% fetal bovine serum (FBS). GSC 8–18 and GSC 7–2, cells were maintained in DMEM/F12 media supplemented with B27 (Invitrogen), EGF (20 ng/ml), bFGF (20 ng/ml) as previously described [49]. For normoxic conditions, cells were incubated in a humidified chamber at 37°C with 5% CO2. Normal neural stem cells were purchased from ThermoFisher Scientific (Cat# N7800100) and grown in media for glioma stem cells described above. Temozolomide(TMZ) was purchased from Sigma-Aldrich (Cat# T2577) and glumatinase (GLS) inhibitor was purchased from EMD Millipore (Compound 968, cat#352010).

### Lactate assay

Equal number of cells were plated in 96 well plates and the culture media were collected after 24 or 48 hours to determine lactate concentration in triplicates with a colorimetric kit following the manufacturer's instructions (Eton Bioscience Inc. San Diego, CA, USA). The absorbance was determined at 490 nm using a plate reader.

### Oxygen consumption assay

Oxygen consumption rate was directly measured using the Instech Fiber Optic oxygen monitor (model 110, Instech Laboratories, Plymouth Meeting, PA) as per the manufacturer's instructions. Cells were trypsinized, counted and 10 million cells were re-suspended in 500 μL of media and warmed in a 37°C water bath. Oxygen tension was measured in triplicates and the rate of oxygen consumption per unit time was calculated and expressed as nmol O_2_/million cells/min.

### Immunohistochemistry (IHC)

Formalin-fixed, paraffin-embedded 5 μm-thick sections were de-paraffinized in xylene and rehydrated in graded ethanol and rinsed in dH_2_O. Heat-induced antigen retrieval was used by pressure-cooking the sections for 20 minutes in citrate buffer (pH = 6). Next, endogenous peroxide activity was blocked in 3% hydrogen peroxide in methanol for 20 minutes. Slides were incubated with primary antibodies in appropriate conditions. Detection was performed using Vectastain ABC reagent and DAB chromogen (Vector Labs, Burlingame, CA). Slides were counterstained in Meyers Haematoxylin for 1 minute; dehydrated through ethanol (70%, 95%, 100% and 100%) and coverslips were mounted using Permount (Fisher). Flash-frozen 5 um sections were air-dried and rehydrated in graded ethanol. Sections were permeabilized in PBS 0.1% Triton X-100 and endogenous peroxidase activity was inhibited by 3% hydrogen peroxide. Slides were blocked with 10% FBS in PBS and incubated with primary antibodies under appropriate conditions. Detection was performed as described above. Primary antibodies used were: MIB-1/Ki67 (Dako), HIF1α (BD Transduction), HK2 (Cell Signaling), CD31 (Millipore), cleaved caspase 3 (Cell signaling). CAIX antibody was kindly provided by Dr. Wouters' laboratory. TUNEL staining was performed with the DeadEnd™ colorimetric TUNEL System (Promega) according to manufacturer's instructions. All of the slides were scanned using Zeiss Mirax scanner and analyzed by Mirax Viewer software.

### Western blotting and antibodies

To analyze protein expression, cells grown on 10 cm plates were scraped off or trypsinized, spun and re-suspended in RIPA lysis buffer supplemented with protease and phosphatase inhibitors (Sigma-Aldrich). Samples were incubated at 4°C for 30 minutes to be lysed sufficiently and then centrifuged at 14,000 rpm for 10 min at 4°C, and the supernatant was collected. Protein concentrations were determined by Bicinchoninic acid (BCA) assay as per the manufacturer's instructions (Pierce Chemical Co., Rockford, IL). A total of 20–30 μg of protein was separated on 8% or 10% SDS-polyacrylamide gel electrophoresis (SDS-PAGE) and blotted onto PVDF membranes (NEN Research Products/Du Pont, Boston, MA) using a semi-dry transfer apparatus (Bio-Rad, Hercules, CA). After blocking the membrane with PBS (or TBS) containing 5% nonfat dry milk and 0.1% Tween 20 (Sigma-Aldrich) for 1 hour, the membranes were incubated with primary antibodies overnight at 4°C or 1 hour at room temperature. The following antibodies were used: β-actin (1:20,000, Sigma-Aldrich), HK2 (1:1,000, Cell Signaling) HK1 (1:1,000, Cell Signaling), ERK1/2 (1:2000, Cell Signaling cat#9102) and Phospho-ERK1/2 (Thr202, Tyr204) (1:2000, Cell Signaling cat#4370). Membranes were washed and then incubated with horseradish peroxidase-conjugated secondary antibodies raised against the corresponding primary antibodies (BioRad, Hercules, CA). Protein bands were detected with Chemiluminescence Reagent Plus (PerkinElmer Inc., Massachusetts, USA). Densitometric analysis was performed using ImageJ software (http://rsbweb.nih.gov/ij/).

### Mouse xenograft studies

All animal procedures were performed in accordance with UHN institutional animal care guidelines. 6–8 weeks old male NOD-SCID mice were used for intracranial xenograft studies. All intracranial injections were performed under general anesthesia (intraperitoneal injections of Avertin 1.25% solution, 0.2 mL/10 g body weight, approximately 0.45 mL for 6 week-old mice). Mice were monitored for any change in their status and were sacrificed according to animal protocol upon reaching a pre-moribund state, which is typically characterized by hunched or abnormal posture, decreased movement, lethargy, paralysis, or weight loss. For intracranial injections, approximately 150,000 cells were suspended in 5 μL PBS and were injected into the right frontal lobe of mice. The head was cleaned with 70% ethanol, the scalp was opened by a 1 cm incision and the landmark was identified for injections (coordinates calculated from bregma). A small hole was drilled over the target area and the cells were injected using a Hamilton syringe.

### Treatment arms

Mice received intracranial injections of gliomas cell lines with conditional HK2 shRNA construct. At 7 days post implantation, mice were imaged by bioluminescence imaging (BLI, see below for detailed methods) to assess baseline tumor presence/size. Mice without tumors or smaller BLI signals than the average were excluded from the study. Mice with comparable BLI measurements were randomized into dox (HK2 KD) and control groups (no dox). At 14 days post intracranial injections mice with established tumors confirmed by BLI were randomized into 2 groups: 1) Radiation alone (2 Gy daily × 3 days – every other day), and 3) concomitant TMZ and radiation (TMZ given two hours before each radiation dose). TMZ was administered by oral gavage.

Mice were followed for survival analysis and were sacrificed when symptomatic. Post-sacrifice, brains were harvested to acquire pathological data. For brain pathology, a group of mice were perfused with ice-cold saline followed by 4% paraformaldehyde (PFA) and the brains were harvested, incubated in PFA overnight followed by snap-freeze in liquid nitrogen or stored at −80°C. Another group of mice were euthanized, and their brains were removed immediately and fixed in formalin, followed by paraffin embedding. For *in vivo* HK2 knockdown experiments, mice were fed sterile doxycycline-containing food pellets.

### Statistical analysis

*In vitro* experiments were performed in triplicates. Means and standard deviations (SD) or standard errors of the means were computed. Student's *t-test* was used for pairwise comparison. ANOVA in combination with Tukey's multiple-comparison post-hoc test was performed for multivariate analysis. Significance was defined at *p < 0.05. On the graphs, asterisks denote a significant difference (*p < 0.05; **p < 0.01; ***p < 0.001) *In vivo* survival analysis was performed using Kaplan-Meier curve generated in Prism 6 (GraphPad), and statistical significance was measured using log-rank (Mantel-Cox) test.

Please see Supplementary Materials and Methods for additional information.

## SUPPLEMENTARY MATERIALS


