# Supplementary material for: Targeting hexokinase 2 enhances response to radio-chemotherapy in glioblastoma
Source: Oncotarget. 2016 Aug 29;7(43):69518–35. doi: 10.18632/oncotarget.11680 (PMC5342495; doi:10.18632/oncotarget.11680)
Supplement: Supplementary file 1 [file oncotarget-07-69518-s001.pdf]

Targeting hexokinase 2 enhances response to radio-chemotherapy in glioblastoma

Supplementary Materials

Transfection of plasmids, siRNA transfection and generation of inducible and luciferase cell lines

The pTRIPZ lentiviral inducible shRNAmir vector system (Open Biosystems) was used containing lentiviral elements, tet-on elements and a puromycin resistance gene. HK2 expression would be conditionally inhibited by two independent small hairpin RNAs (shRNAs) against HK2 and a control scrambled shRNA. Addition of doxycycline (Dox, tetracycline derivative) induces expression of shRNAmir and turbo red fluorescent protein (turboRFP) in the pTRIPZ vector. shRNA and RFP reporter are expressed from the same transcript under the control of a tetracycline response element (TRE) promoter, which allows for quick assessment of viral titer, transduction efficiency, and level of knockdown *in vitro* and *in vivo*. Lentiviral particles were generated by co-transfection of HEK293T (human embryonic kidney) cells with third generation packaging plasmids, one encoding the vesicular stomatitis virus G envelope glycoprotein, one encoding gag-pol genes, and the third being the respective shRNA lentiviral vector. The supernatants containing the lentiviral particles were harvested 48 and 72 hours post-transfection, centrifuged at 2,000 rpm for 10 minutes and then filtered through a 0.22 μm sterile filter. Cells were plated at a density of 100,000 cells/well in a 24-well tissue culture plate in antibiotic-free medium one day prior to transduction. At the time of transduction, lentivirus was added to the cells as per instructions for pTRIPZ and incubated at 37°C for 12–24 hours. The medium was then removed and replaced with new media for another 48 hours. To select for stably transduced cells, medium containing puromycin (1 μg/mL) was added 72 hours post-transduction. To assess the transduction efficiency following infection or to induce shRNA expression, 1 μg/mL dox was added to the media. 48 hours post Dox treatment, the proportion of cells expressing RFP were determined using a fluorescence microscope to approximate the transduction efficiency. Western blot analysis was performed to quantify the level of knockdown in three independent shRNA constructs. For knockdown conditions, cells were maintained in puromycin (1 μg/mL) and dox (1 μg/mL) throughout the experiment.

U87 cell lines were transduced with a lentiviral vector containing a firefly luciferase (F-luc) gene under the control of cytomegalovirus (CMV) promoter. The lentiviral luciferase vector also contains a green fluorescent protein (GFP) reporter gene. GS2 cell lines were already modified with a lentiviral vector containing firefly luciferase (48). Viral particles were harvested from supernatant of cells 48 and 72 hours post-transfection and were used to infect U87 cells. GFP-positive cells were purified and sorted by FACS (transfection efficiency > 90%). GFP-positive cells were lysed, treated with luciferin (D-luciferin potassium salt, 150 mg/kg, Gold Biotechnology, St Louis, MO) and a luciferase assay was performed. Luciferase levels were quantified using a luminometer and values (expressed as Relative Light Units) were normalized to total protein content of each sample. ERK2 rescue experiments were performed by electroporation of a constitutively active ERK2 plasmid: pWZLblasti-HA-ERK2 R67S which was a gift from Christopher Counter (Addgene plasmid # 53172). Electroporation of 4 ug of ERK2 active plasmid using Neon Transfection System and manufacturers protocol (Invitrogen CA, USA, Cat# MPK5000) was performed in U87 and GSC8-18 HK2 inducible knockdown cells. Cell viability in ERK2 R67S rescue experiments were assessed by Almar Blue Cell Viability Dye and the manufacturers protocol (ThermoFisher Scientific USA, Cat#DAL1025).

Cell growth and comet assays

Cell viability was measured by direct cell count using the Vi-CELL Coulter Analyzer (Beckman). 50,000 to 80,000 cells were seeded into 6 well plates and incubated for up to 6 days. Cells were trypsinized and counted in triplicates at days 2, 4, and 6 post-incubation. DNA damage was visualized with comet assay using Trevigen method (Trevigen, INC, Helgerman CT. Cat#4250-050-01). Cells were trypsynized and counted. Next,the cells were pelleted, washed once in ice-cold 1× PBS, resuspended at 1×10^5 cells/ml in ice cold PBS and placed on ice. In treatment conditions, cells were kept on ice before lysis. Cells were combined with molten low melt agarose (LMAgarose) at 37°C at a ratio of 1: 10 (v/v) and 100 μL was immediately pipetted onto a CometSlideTM. Slides were placed flat at 4°C in the dark for 30 minutes and then immersed in pre-chilled lysis solution at 4°C for additional 45 minutes. Slides were treated with alkaline

solution, pH > 13, for 60 minutes at room temperature in the dark to unwind and denature the DNA. Next, slides were transferred to a horizontal electrophoresis apparatus containing alkaline electrophoresis solution and the power was set to 1 Volt. After 20 minutes, the slides were removed, dipped in 70% ethanol, air-dried and stored with desiccant at room temperature. For scoring, the slides were stained with 1× ethidium bromide for 25 minutes, coverslips were added and the slides were scored immediately by epifluorescence microscopy. For each sample more than 50 comets were scored and analyzed. The comet parameter considered by the software in this study was the Olive tail moment, which is the product of percent tail DNA and the distance between the head and the tail along the x-axis of the comet.

Bioluminescence imaging (BLI)

Bioluminescence imaging (BLI) was conducted using the Xenogen IVIS system of the Xenogen 100 series coupled with Living Image software for data acquisition (Xenogen Corp.). Mice were administered luciferin (D-luciferin potassium salt, 150 mg/kg, Xenogen Corp.) via IP injection and were anesthetized with isoflurane after 4 minutes and serial images were obtained following injections. The time of peak distribution within each group of mice was determined by serial image acquisition. For BLI signal analysis, Living Image software was used to define fixed regions of interest surrounding the intracranial area of signal and the total number of photons per second per steradian per square centimeter was recorded.

Radiation

In vitro irradiation of cells was done using a Gamma Cell 40 irradiator (Nordion International) with a dose rate of 1 Gy/min for a total of 4 Gy. For *in vivo* radiation treatments, mice were anesthetized using isoflurane and placed in an in-house custom-built immobilization device. Stereotactic irradiation was delivered using a cone-beam CT image-guided small animal irradiator (XRad 225Cx micro-IGRT unit, Precision X-Ray, Inc). Radiation was prescribed with anterior-posterior parallel-opposed beams using a 1cm collimator and irradiation was guided to the right cortex of the brain. Fractionated radiation schedule was used, delivering 6 Gy in 3 fractions of 2 Gy every other day.

Hexokinase activity assay and ELISA

Hexokinase activity assay was purchased from BioVision, Milpitas, California, USA (cat# K789-100) and performed per manufacturers protocol. HK2 ELISA assay was purchased from MyBioSource, San Diego, California, USA (cat# MBS726852) and performed as per manufacturers protocol.

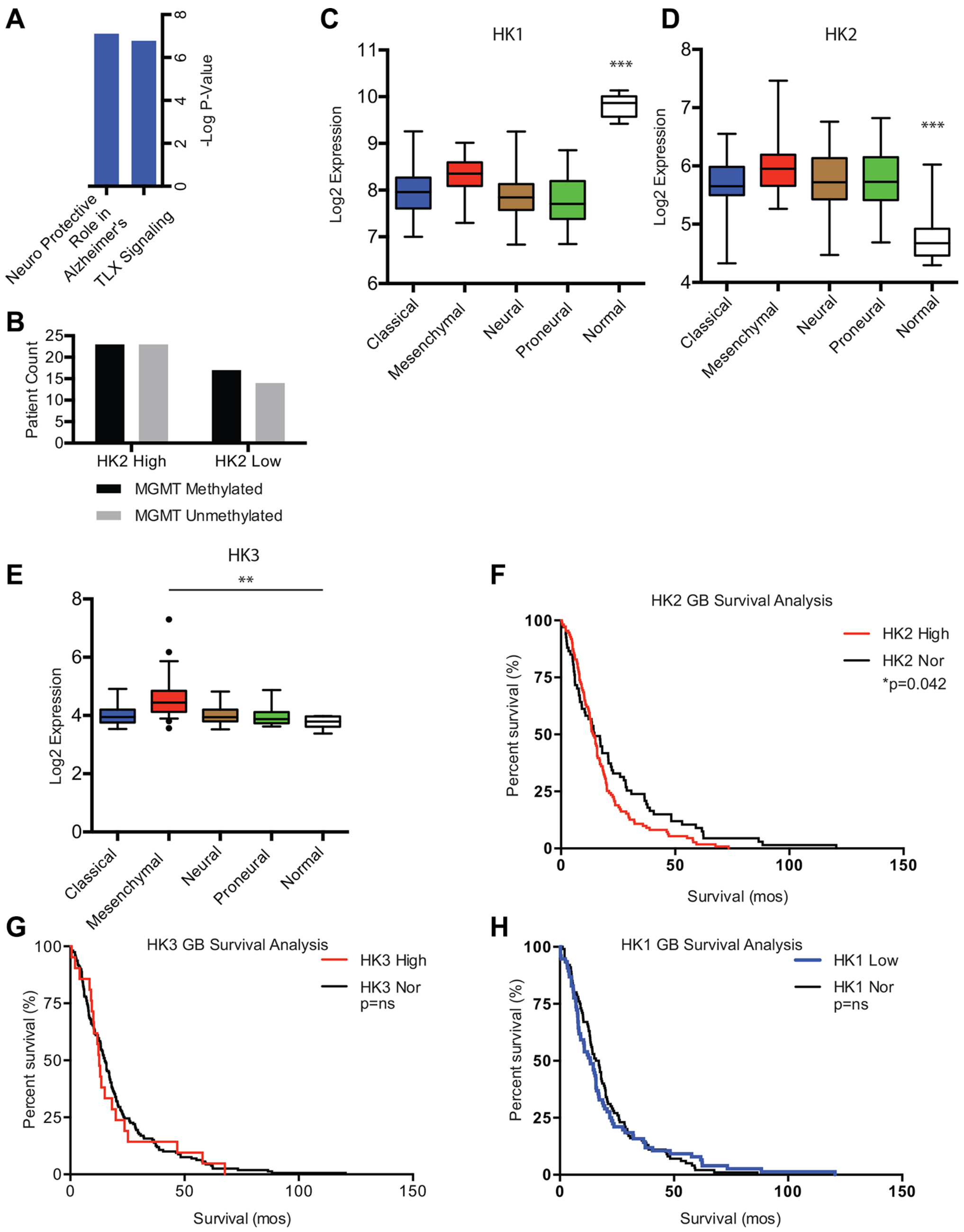

Supplementary Figure S1: HK2 but not HK1 or HK3 is of prognostic value. (**A**) Ingenuity pathway analysis of signaling networks inactivated in high HK2 expressing GBM patients. (**B**) MGMT promoter methylation status between high and low HK2 expressers. (**C**) HK1 is downregulated at the mRNA level in all GB subtypes compared to normal brain. (**D**) HK2 is upregulated at the mRNA level in all GB subtypes compared to normal brain. (**E**) HK3 is upregulated at the mRNA level in all GB subtypes compared to normal brain. (**F**) Patients with high HK2 at the mRNA level (> 2 fold compared to normal brain) in GB have a poorer overall survival in the REMBRANDT GB data set. (**G**) Patients with high HK3 at the mRNA level (> 2 fold compared to normal brain)in GB show no survival in the REMBRANDT GB data set. (**H**) Patients with low HK1 in GB have no survival benefit at the mRNA level (< 2 fold compared to normal brain) in the REMBRANDT GB data set. $^{*}p < 0.05$, $^{**}p < 0.01$; $^{***}\ p < 0.001$.

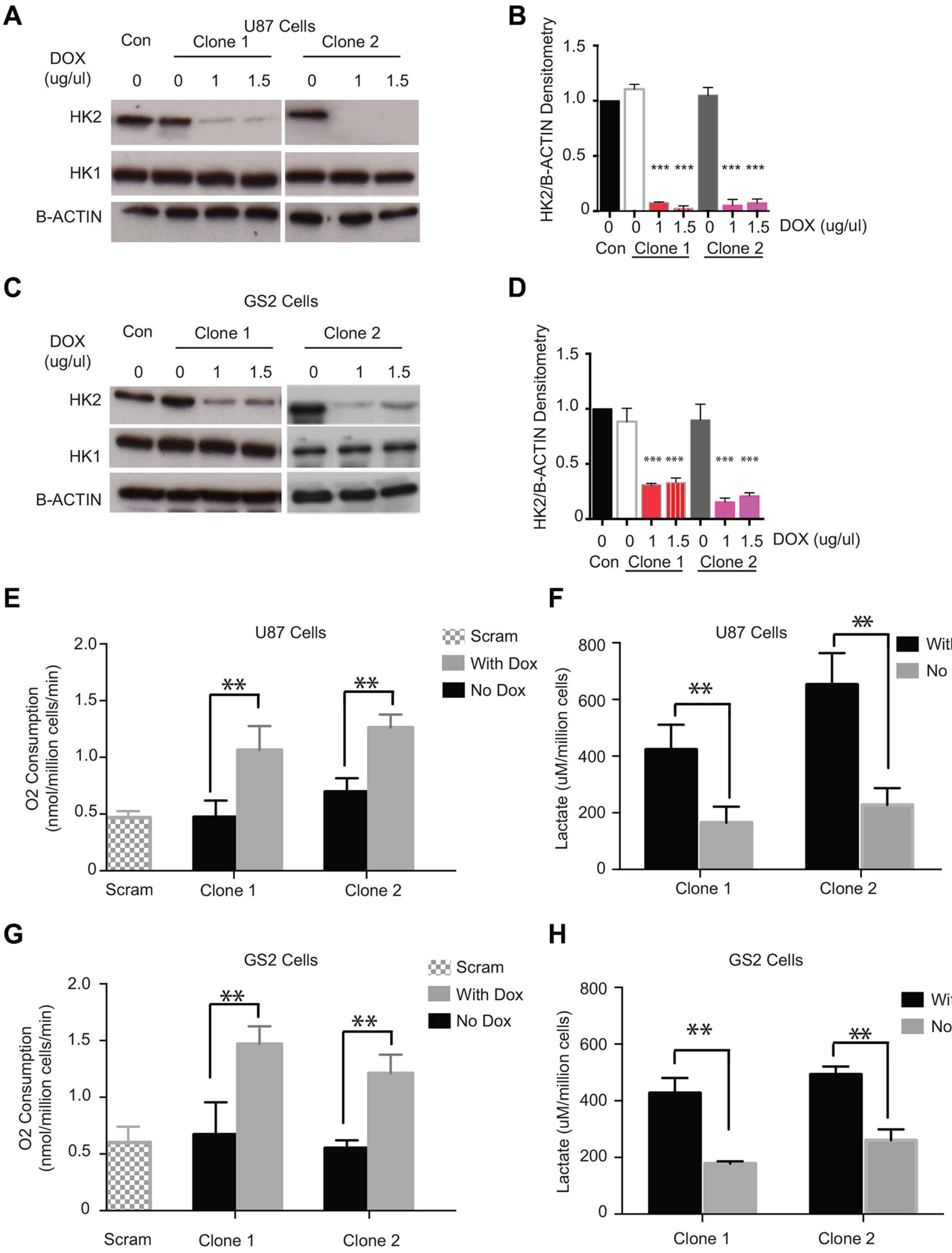

Supplementary Figure S2: Generation of inducible HK2 KD GB cells. (**A**) Western blot analysis of inducible HK2 shRNA constructs in U87 cells in the presence of varying amounts of doxycycline (Tet-On system). Two U87 HK2 KD clones were selected and compared to control cells (U87 cells expressing non-targeting shRNA). (**B**) Densitometric analysis of HK2 in control cells and in HK2 shRNA inducible cells treated with varying amounts of doxycycline from A. (**C**) Western blot analysis of inducible HK2 shRNA constructs in GS2 cells treated with varying amounts of doxycycline (Tet-On system). Two GS2 HK2 KD clones were selected and compared to control cells (GS2 cells expressing non-targeting shRNA). (**D**) Densitometric analysis of HK2 in control cells and in HK2 shRNA inducible cells treated with varying amounts of doxycycline from C. (**E**) Oxygen consumption of control U87 (scram) and U87 clones 1–2 expressing HK2 shRNA in the presence of doxycycline (HK2 KD) or absence of doxycycline (HK2 expressed). O2 consumption was normalized per million cells. (**F**) Lactate production of control U87 (scram) and U87 clones 1–2 expressing HK2 shRNA in the presence of doxycycline (HK2 KD) or absence of doxycycline (HK2 expressed). (**G**) Oxygen consumption of control GS2 (scram) and GS2 clones 1–2 expressing HK2 shRNA in the presence of doxycycline (HK2 KD) or absence of doxycycline (HK2 expressed). O2 consumption was normalized per million cells. (**H**) Lactate production of control GS2 (scram) and U87 clones 1–2 expressing HK2 shRNA in the presence of doxycycline (HK2 KD) or absence of doxycycline (HK2 expressed).

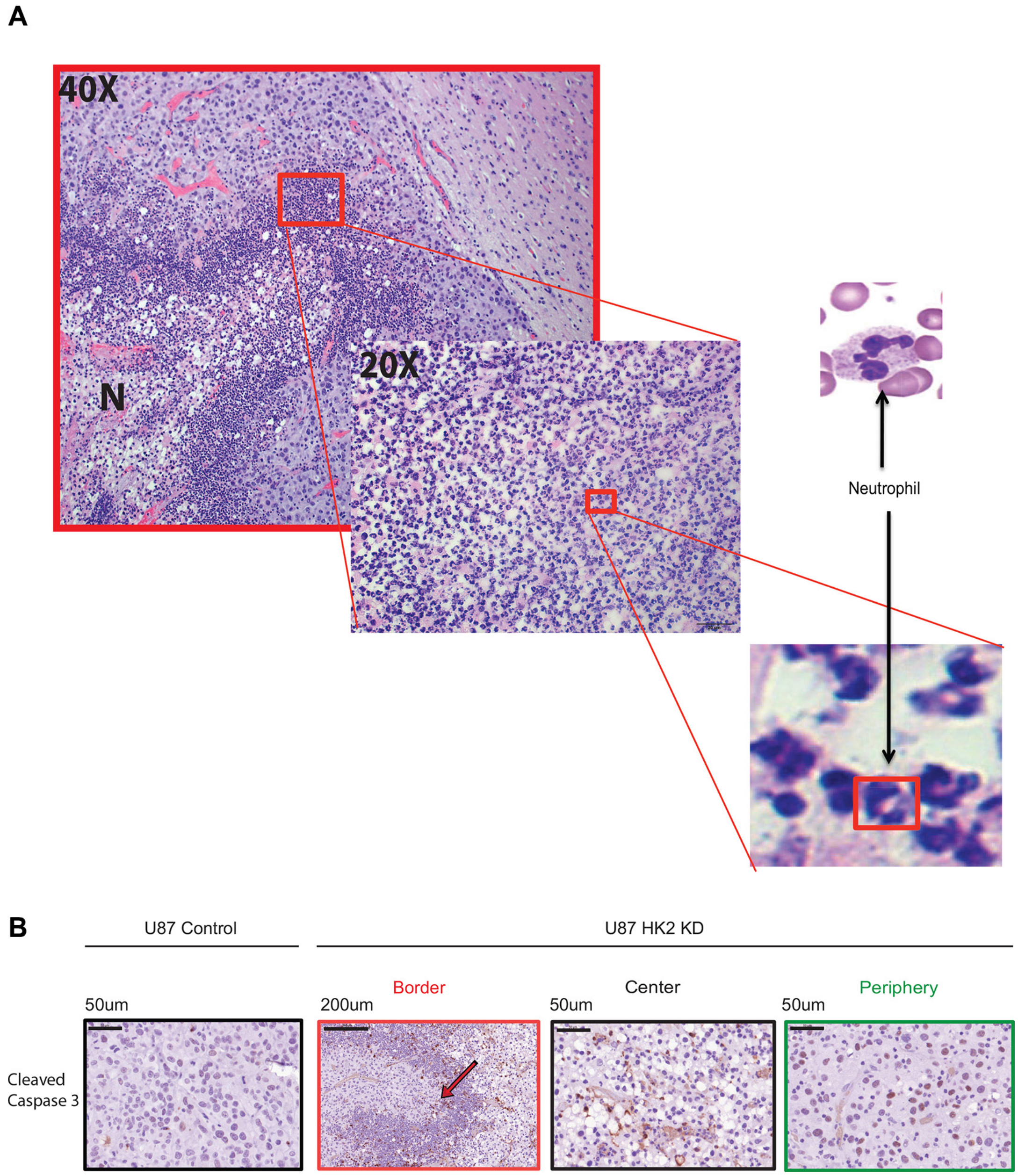

Supplementary Figure S3: Neutrophil recruitment to HK2 knockdown tumors. (**A**) Close up of infiltrating neutrophils at the border region of U87 HK2 KD tumors. Infiltrating neutrophils were not detected in control tumors. (**B**) IHC analysis of Cleaved Caspase 3, a measure of apoptosis in U87 control and U87 HK2 KD tumors (dox treated).

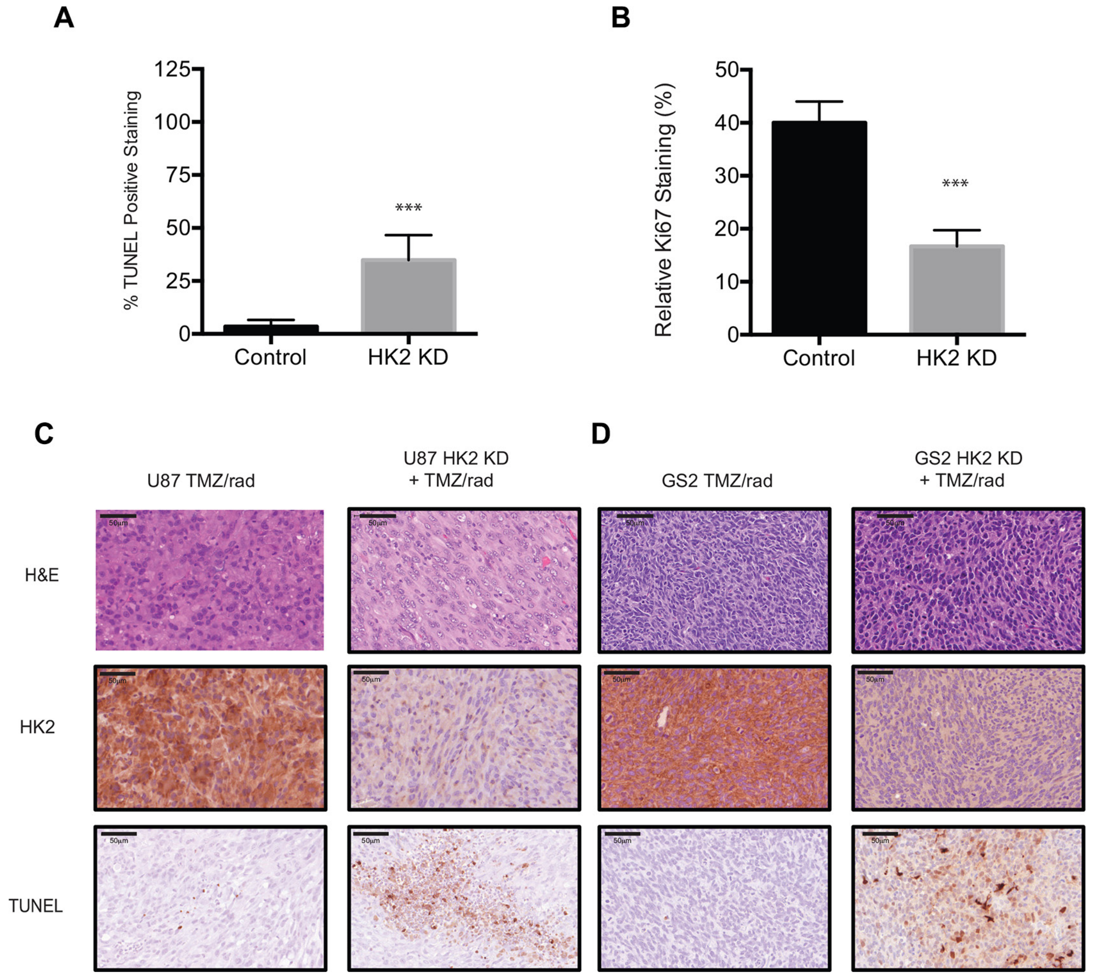

Supplementary Figure S4: (A) TUNEL staining was performed on 5 mice and 20 fields of view per mouse at 200X total magnification were used to determine %positive TUNEL cells. (**B**) Ki67 quantification from U87 xenografts and U87 HK2 KD xenografts. (**C**) Representative H&E and IHC of HK2 and TUNEL staining in U87 treated with RAD/TMZ (left) and HK2 KD U87 treated with RAD/TMZ (right) mice. Scale bar = 50 µm. (**D**) Representative H&E and IHC of HK2 and TUNEL staining in GS2 RAD/TMZ (left) and HK2 KD GS2 treated with RAD/TMZ (right) mice. Scale bar = 50 µm. ***$p < 0.001$.
